# Endoparasites of marsupials in fragments of the Atlantic rainforest, western Paraná State, Brazil

**DOI:** 10.1590/S1984-29612023056

**Published:** 2023-10-13

**Authors:** Danise Benatti, Marcela Figueirêdo Duarte Moraes, Carmen Andrea Arias Pacheco, Dália Monique Ribeiro Machado, Wilson Junior Oliveira, Patricia Parreira Perin, Luís Felipe Andrietti, José Flávio Cândido, Alexandre Vogliotti, José Hairton Tebaldi, Estevam Guilherme Lux Hoppe

**Affiliations:** 1 Laboratório de Enfermidades Parasitárias - LabEPar, Departamento de Patologia, Reprodução e Saúde Única - DPRSU, Faculdade de Ciências Agrárias e Veterinárias - FCAV, Universidade Estadual Paulista - UNESP, Jaboticabal, SP, Brasil; 2 Universidade Estadual do Oeste do Paraná - UNIOESTE, Cascavel, PR, Brasil; 3 Universidade Federal da Integração Latino-Americana - UNILA, Foz do Iguaçu, PR, Brasil

**Keywords:** Didelphimorphia, habitat fragmentation, helminths, neotropical, Didelphimorphia, fragmentação de habitat, helmintos, neotropical

## Abstract

Knowledge of taxonomy and biodiversity of parasites is fundamental to better understand ecosystem dynamics. The objective of this study was to describe the helminth fauna of two species of marsupials in five fragments of the Atlantic rainforest in the western region of Paraná State, Brazil. In a total of 4050 trap-nights, the animals were captured using Sherman, Tomahawk, and Pitfall traps, euthanized, necropsied, and their organs inspected for helminths. After identification of the parasites, descriptors of infection, such as prevalence, mean abundance, mean intensity, and range of intensity, were calculated. Collectively, six helminth species were observed in 18 animals. The following five species were observed in *Marmosa paraguayana: Viannaia hamata* (58.8%), *Gracilioxyuris agilisis* (52.9%), *Travassostrongylus sextus* (17.6%), *Oncicola luehei* (5.9%), and *Pritchardia boliviensis* (5.9%). Whereas the following two species were observed in *Monodelphis dimidiata: Trichohelix tuberculata* (100%) and *Travassostrongylus sextus* (100%). This study represents a new locality record for all helminths described herein, and a new host for four helminth species. This is the first report on the helminth fauna of *Monodelphis dimidiata*, expanding knowledge about marsupials in the Brazilian Atlantic Forest.

## Resumo

Conhecimentos taxonômicos e da biodiversidade parasitária são fundamentais para uma melhor compreensão da dinâmica do ecossistema. O objetivo do estudo foi descrever a helmintofauna de marsupiais em cinco fragmentos de Mata Atlântica, na região Oeste do Estado do Paraná, Brasil. Em um total de 4.050 armadilhas/noite, os animais foram capturados com as do tipo Sherman, Tomahawk e Pitfall, eutanasiados, necropsiados e seus órgãos inspecionados em busca de helmintos. Após a identificação do parasita, foram calculados os indicadores de infecção (prevalência, abundância média, intensidade média e variação de intensidade). Seis espécies de helmintos foram observadas em dezoito animais; cinco em *Marmosa paraguayana: Viannaia hamata* (58,8%), *Gracilioxyuris agilisis* (52,9%), *Travassostrongylus sextus* (17,6%), *Oncicola luehei (5,9%)* e *Pritchardia boliviensis* (5,9%), e duas em *Monodelphis dimidiata: Trichohelix tuberculata* (100%) *e Travassostrongylus sextus* (100%). Este estudo representa um novo registro de localidade para todos os helmintos descritos e um novo hospedeiro para quatro deles. Trata-se do primeiro relato da helmintofauna de *Monodelphis dimidiata*, expandindo-se os conhecimentos sobre marsupiais na mata Atlântica brasileira.

## Introduction

Knowledge of the parasite biodiversity in the context of taxonomic studies is fundamental for a better understanding of ecosystem dynamics; however, this field of research has been neglected for a long time (Poulin, 2007). Parasites are organisms that are critical for the maintenance of ecosystems and assist in the regulation and structuring of the host population, interfering with processes, such as competition, migration, reproduction, and speciation (Marcogliese, 2005). Understanding the processes that regulate these interactions will reveal the structure and dynamics of parasite-host interactions (Poulin, 2010, 2013, 2021) and its role as an etiological agent and, consequently, its potential risk to public and animal health (Martins & Bonato, 2004).

In South America, the order Didelphimorphia is represented by the family Didelphidae, which includes 18 genera and 91 species (Gardner, 2008). In Brazil, the family is represented by 16 genera and 62 species, which are present in all biomes, with most species in the Atlantic and Amazon rainforests (Cáceres, 2013; Faria et al., 2019; Quintela et al., 2020; Cáceres & Dickman, 2022). Most parasitological studies on Brazilian marsupials have focused on *Didelphis* spp. (Cirino et al., 2022; Freitas et al., 2022). At present, 20 digeneans, three cestodes, 55 nematodes, and four acanthocephalans are known to parasitize these mammals (Vicente et al., 1997; Quintão & Costa, 1999; Noronha et al., 2002; Gomes et al., 2003; Boullosa et al., 2017; Simões et al., 2017; Zabott et al., 2017; Costa-Neto et al., 2019). However, most of the studies are limited to parasite descriptions and lack data on the structure and variations of helminths that infects marsupials (Freitas et al., 2022)

The Atlantic rainforest is the second largest tropical rainforest on the American continent, after the Amazon rainforest. It is considered one of the most endangered and crucial for conservation biomes in the world (Marques et al., 2021). Due to the expansion of human activities, the Atlantic rainforest has suffered from fragmentation processes over the years, and its native vegetation has reduced from 16% to 11.4% of its original area. Moreover, only 7% of the remaining fragments of the Atlantic rainforest have a total area larger than 100 acres (Myers et al., 2000; Tabarelli et al., 2005; SOS Mata Atlântica, 2019).

Therefore, this study aims to describe the helminths that colonize two marsupial species, *Marmosa paraguayana* and *Monodelphis dimidiata* in five small fragments of the Atlantic rainforest in the western region of Paraná state, Brazil.

## Material and Methods

### Study area and animals

This study was conducted in fragmented areas of the Atlantic rainforest located in the western portion of Paraná State, near the municipalities of Cascavel (24°57'21”S 53°27'18”W) and Corbélia (24°47'56”S 53°18'25”W) in Brazil. The climate in these regions is humid subtropical with an average annual temperature of approximately 19 °C. The characteristic vegetation of these regions is that of a semi-deciduous seasonal forest (IBGE, 2017). These small fragments are interspersed in a strongly anthropized matrix surrounded mainly by agricultural activity, especially soy and corn crops (Ribeiro et al., 2009).

**Figure 1:**
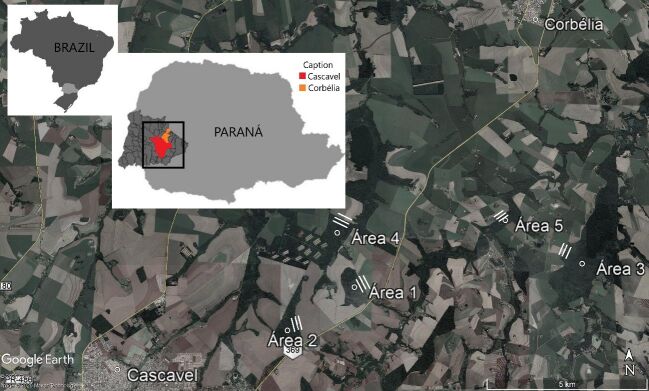
Capture points of *Marmosa paraguayana* and *Monodelphis dimidiata* in the study area. The points are numbered from 1 to 5 (Circles) between Cascavel and Corbélia cities (Paraná state, Brazil). Grey area: Paraná state. Dark grey area: Western region of Paraná State. Red area: Cascavel municipality. Orange area: Corbélia municipality.

Sherman (32.4 × 11.7 × 14.2 cm) (Sherman Traps Inc., Tallahassee, FL), Tomahawk (29.5 × 11 × 10.5 cm) (Tomahawk Live Trap Co., Tomahawk, Wis), and Pitfall (60-liter buckets and canvas barrier 50 cm high by 18 m long) traps were used for the samples, following the recommendation of Cáceres (2013). The traps were established in five areas of 3.6 acres, composed of three parallel trapping lines, with the central line positioned at the interface of the fragment with the adjacent agricultural matrix and the outer lines 100 m towards the interior of the fragment and cultivated area (Figure 1). Each line contained 10 traps placed 20 m apart and a pit-fall line. The traps were baited with pineapple slices and a mixture of paçoca (a Brazilian candy made of crushed peanut and sugar), oats, grated tuna, and bacon. The traps were inspected every morning for animals and the baits were renewed (Mangini & Nicola, 2006). Sampling was conducted in campaigns of five consecutive nights, simultaneously, in September, November, and December of 2017 and January and February of 2018, which corresponded to the initial and late stages of soy cultivation in the study area, and April, June, July, and August of 2018, which corresponded to the initial and late stages of corn cultivation in the study area, for a total of 4050 trap-nights.

The captured animals were transported to the Laboratory of Metabolism and Human and Animal Physiology at Western Paraná State University (Unioeste-Cascavel Campus, Cascavel, Brazil), where they were euthanized using isoflurane according to the standards estabilished by the Federal Council of Veterinary Medicine. Taxonomic identification was based on analysis of guard hairs, and external and cranial morphology, as described previously by Cáceres (2013) and Faria et al. (2019). The sex, weight, body length, tail length, hind limb length, ear length, and body width of each animal was recorded according to the protocol described by Reis et al. (2010). The animals were then eviscerated, and all cavitary organs and diaphragms were removed and stored in identified dry flasks at −20 °C. These samples were sent to the Laboratory of Parasitic Diseases of the School of Agricultural and Veterinarian Studies of São Paulo State University (LabEPar, FCAV/Unesp, São Paulo, Brazil) for parasitological assessment.

### Parasitological analysis

The anatomical segments of the digestive tract (esophagus, stomach, small intestine, and large intestine), as well as the omentum, mesentery, trachea, heart, lungs, liver, spleen, and kidneys, were separated, slits were made if necessary, and the organs were carefully inspected under a stereoscopic microscope to search for helminths. The observed helminths were collected and fixed in a 70% ethanol solution and stored in small flasks to identify the host as well as the site of infection.

The helminths apart from *Pritchardia boliviensis* were clarified using an 80% acetic acid solution, following the method described by Travassos (1950). *Pritchardia boliviensis* was submitted to a regressive process of carmine staining (Amato & Amato, 2010). Taxonomic identification was based on 10 adult individuals of each sex for dioecious and monoecious species, or the maximum number available for the species, in which case the number was stated. Morphological characteristics were assessed on temporary mounts using an Olympus BX-51 microscope (Olympus, Tokyo, Japan) attached to a Q-Color 3 digital camera (Olympus, Tokyo, Japan), and the images were processed using Image-Pro Plus 4 image analyzer software (Media Cybernetics, Rockville, MD, USA). Taxonomic identification was based on the reports of Travassos (1917), Yamaguti (1963), Rêgo (1967), Vicente et al. (1997) and Anderson et al. (2009). Vouchers were deposited in the Collection of Oswaldo Cruz Institute (CHIOC accession numbers: 38792, 38788, 38789, 38903, 38905, and 38907), and additional specimens were kept in LabEPar’s helminthological collection.

### Data analysis

Descriptors of infection (prevalence, mean abundance, mean intensity, and range of intensity) were calculated after identification and counting of the parasites according to the protocol reported by Bush et al. (1997). The host body condition index (BCI) was determined by the relationship between mass (g) and total length (cm) using the following equation: BCI = mass (g)/total length (cm) (Schulte-Hostedde et al., 2005). Prior to statistical analysis, the Kolmogorov-Smirnov test revealed non-normal data sets; therefore, the non-parametric tests were used for statistical analysis. To assess whether the sex of the hosts influences the prevalence of each helminth, Fisher's exact test was used. Nonlinear regression analysis was used to investigate the influence of total parasite intensity on BCI. All tests were performed using GraphPad Prism 7.04 software (GraphPad Software Inc., San Diego, CA, USA) with the p-value adjusted to 0.05. Because only one individual of *M. dimidiata* was captured, statistical analyses could not be performed for this species.

## Results

We captured 18 marsupials represented by two species of the order Didelphimorphia: 17 *M. paraguayana* specimens and one specimen of *M. dimidiata*. The fragments where the marsupials were captured, sex and the helminths identified are summarized in Table 1. Fifteen marsupials were parasitized by at least one species of helminth, and 1748 helminths were recovered.

**Table 1:** Distribution of *Marmosa paraguayana* and *Monodelphis dimidiata* captured in five fragmented areas of the Atlantic rainforest in the western region of Paraná state, Brazil, according to area of capture, sex of the marsupials and helminths identified.

ID	Host	Areas	Sex	Diagnosed helminths
5	*Monodelphis dimidiata*	2	M	*Trichohelix tuberculata Travassostrongylus sextus*
27	*Marmosa paraguayana*	3	M	*Gracilloxyuris agilisis*
34	*Marmosa paraguayana*	1	M	*Viannaia hamata*
39	*Marmosa paraguayana*	1	F	*Viannaia hamata*
				*Travassostrongylus sextus*
40	*Marmosa paraguayana*	2	M	*Gracilloxyuris agilisis*
41	*Marmosa paraguayana*	4	M	*Gracilloxyuris agilisis*
43	*Marmosa paraguayana*	4	F	Negative
57	*Marmosa paraguayana*	4	F	Negative
61	*Marmosa paraguayana*	1	M	*Viannaia hamata*
				*Travassostrongylus sextus*
63	*Marmosa paraguayana*	1	M	*Gracilloxyuris agilisis*
				*Viannaia hamata*
69	*Marmosa paraguayana*	3	F	Negative
71	*Marmosa paraguayana*	3	M	*Gracilloxyuris agilisis*
				*Pritchardia boliviensis*
72	*Marmosa paraguayana*	2	F	*Gracilloxyuris agilisis*
				*Viannaia hamata*
74	*Marmosa paraguayana*	2	M	*Gracilloxyuris agilisis*
				*Viannaia hamata*
75	*Marmosa paraguayana*	2	M	*Viannaia hamata*
87	*Marmosa paraguayana*	5	M	*Viannaia hamata*
				*Oncicola luehei*
91	*Marmosa paraguayana*	2	M	*Gracilloxyuris agilisis*
				*Viannaia hamata*
				*Travassostrongylus sextus*
93	*Marmosa paraguayana*	2	F	*Gracilloxyuris agilisis*
				*Viannaia hamata*

**Figure 2:**
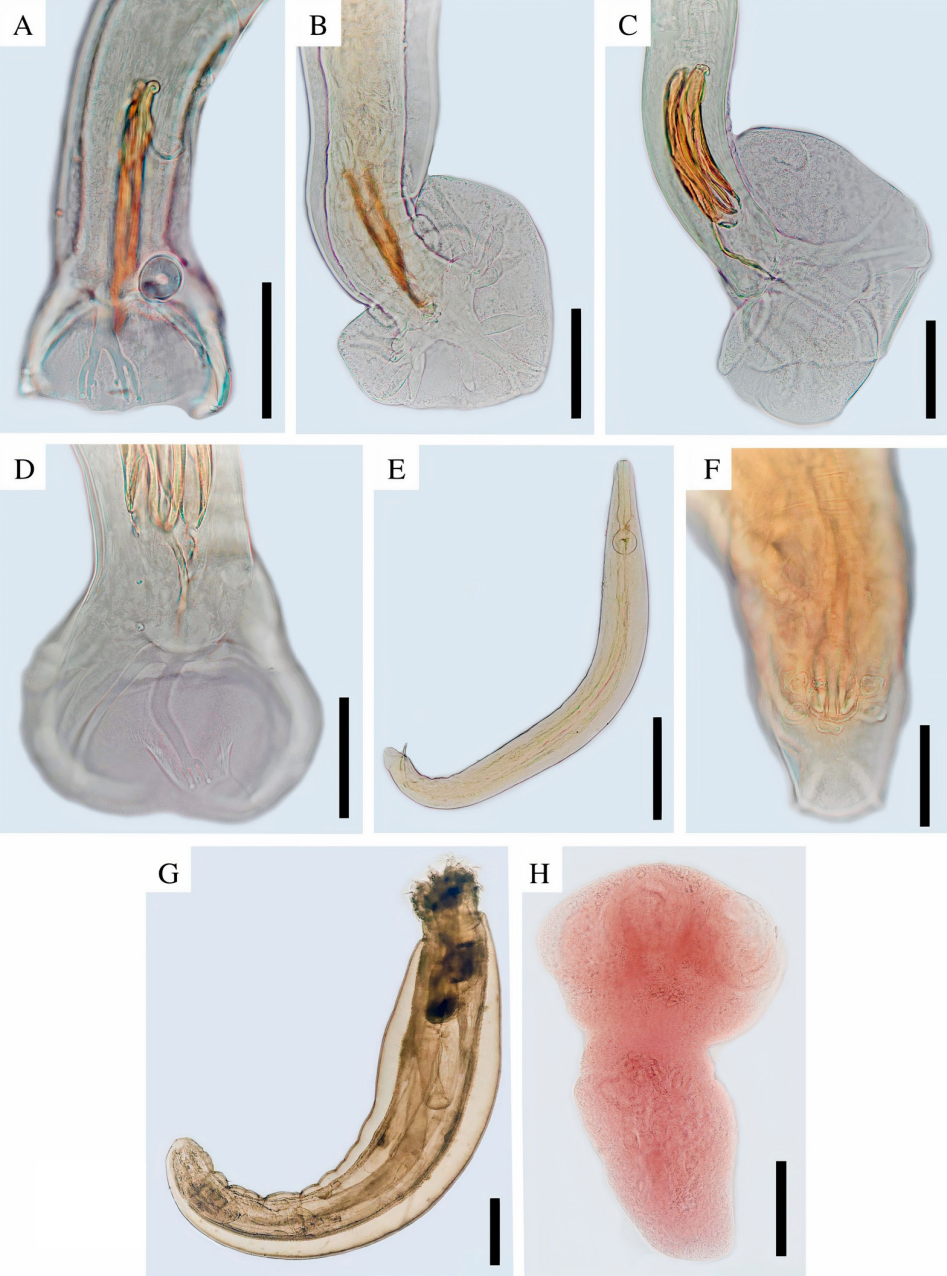
Morphological aspects of the helminths found in *Marmosa paraguayana* and *Monodelphis dimidiata* captured in five fragmented areas of the Atlantic rainforest in the western region of Paraná state, Brazil. A) *Trichohelix tuberculata*, posterior end of a male specimen. Scale bar: 50 µm; B) *Viannaia hamata*, posterior end of a male specimen. Scale bar: 50 µm; C) *Travassostrongylus sextus*, posterior end of a male specimen. Scale bar: 100 µm; D) *T. sextus,* copulatory bursa and dorsal ray. Scale bar: 50 µm; E) *Gracilioxyuris agilisis.* whole view of an adult male specimen. Scale: 200 µm; F) *Gracilioxyuris agilisis*, posterior end of a male specimen, showing the caudal papillae. Scale bar: 50 µm; G) *Oncicola luehei.* Whole view of an adult female specimen. Scale: 200 µm; H) *Pritchardia boliviensis*, scolex. Scale:100 µm.

From the 17 individuals of *M. paraguayana* examined, 14 (83%) were infected by at least one individual helminth. The most frequent helminth species in *M. paraguayana* was the trichostrongylid *Viannaia hamata* (Travassos, 1914) (58.82%, 10/17), followed by the oxyurid *Gracilioxyuris agilisis* (Feijó et al., 2008) (52.94%, 9/17). *Pritchardia boliviensis* (Gardner et al., 2013), and *Oncicola luehei* (Travassos, 1917) were identified in 5.88% (1/17) of the analyzed *M. paraguayana*. Two trichostrongylid species, *Travassostrongylus sextus* (Travassos, 1937) and *Trichohelix tuberculata* (Parona & Stossich, 1901), were found in the small intestine of the single *M. dimidiata* specimen (Figure 2, Table 2).

**Table 2:** Site of infection, abundance, mean intensity of the helminths with standard deviation and range of intensity, and helminth prevalence with 95% confidence interval found in *Monodelphis dimidiata* and *Marmosa paraguayana* captured in five fragmented areas of the Atlantic rainforest in the western region of Paraná state, Brazil.

Helminths	Site of infection	*Marmosa paraguayana*			*Monodelphis dimidiata*		
**Cestoda**		Prevalence % (95%IC)	Abundance	Mean intensity ± SD (Range of intensity)	Prevalence % (95%IC)	Abundance	Mean intensity (Range of intensity)
**Anoplocephalidae**							
*Pritchardia boliviensis* (CHIOC 38789)	Small intestine	5.88 (1.04 - 26.98)	46	782 (-)	-	-	-
**Nematoda**							
**Vianaiidae**							
*Viannaia hamata* (CHIOC 38907)	Small intestine	58.82 (36 - 78.38)	3.3	5.7 ± 3.49 (1-12)	-	-	-
*Travassostrongylus sextus* (CHIOC 38903)	Small intestine	17.64 (6.19 - 41.02)	0.3	1.66 ± 0.57 (1-2)	100 (20.65 - 100)	63	63 (-)
**Molineidae**	Small intestine	-	-	-	100 (20.65 - 100)	79	79 (-)
*Trichohelix tuberculata* (CHIOC 38905)							
**Oxyurida**							
**Oxiuridae**							
*Gracilioxyuris agilisis* (CHIOC 38792)	Cecum	52.94 (30.96 - 73.83)	44.7	84.44 ± 67.39 (4-211)	-	-	-
**Acanthocephala**							
**Archiachanthocephala**							
*Oncicola luehei* (CHIOC 38788)	Small intestine	5.88 (1.04 - 26.98)	0.12	2(-)	-	-	-

SD - Standard deviation.

The cestode *Pritchardia boliviensis* and the oxyurid *Gracilioxyuris agilisis* presented a higher mean parasitic abundance and intensity, whereas *T. sextus* and *O. luehei* were less abundant (Table 2). The body condition index (BCI) of *Marmosa paraguayana* varied from 03 to 06, and there was no significant relationship (p > 0.05) between the BCI and total parasite intensity due to the small sample size. Sex hosts significant influenced (p < 0,05) the parasites prevalence, with male prevalence higher than female (Table 3).

**Table 3:** Fisher's exact test results for comparison of parasite prevalence between sex hosts and nonlinear regression analysis to evaluate the association between total parasite intensity and body condition index (BCI) of *Marmosa paraguayana* captured in five fragmented areas of the Atlantic rainforest in the western region of Paraná State, Brazil.

**Analyses**	**Test**		**P**
Interaction of host sex X Parasite prevalence	Fisher's exact	CI 95% = 1.42 - 61.26	0.0133
Body Condition Index X Total Parasite Intensity	Nonlinear regression	F = 1.11	0.4223

## Discussion

Didelphids (Didelphimorphia: Didelphidae) are a large and well-studied group of neotropical marsupials (Gardner, 2008). Consistent with the findings of the present study, several studies have previously suggested that neotropical marsupials are frequent hosts for helminths (Gomes et al., 2003; Torres et al., 2007, 2009; Jiménez et al., 2008; Byles et al., 2013; Chero et al., 2017).

*Marmosa paraguayana* is an omnivorous/insectivorous species that inhabits forest habitats (Cáceres, 2013; Gardner, 2008). Reports of parasites in this host include the cestode *Mathevotaenia bivittata,* found in Argentina (Campbell et al., 2003), as well as the nematodes *Gracilioxyuris agilisis* (Nematoda: Oxyuridae), found in the Brazilian Pantanal wetlands (Santos-Rondon et al., 2012); *Aspidodera raillieti, Viannaia hamata,* and *Trichuris* sp. (Nematoda: Trichuridae), reported in the Brazilian Atlantic Forest (Gentile et al., 2022); *Litomosoides* barretti (Nematoda: Filaroidea), found in Bahia state, Brazil (Muller, 1980); and *Paucipectines elegans* (Nematoda: Rictulariidae), described in Sao Paulo, Brazil (Travassos, 1928). *Gracilioxyuris agilisis* represents the fourth oxyurid genus that infects Neotropical marsupials (Feijó et al., 2008) and the finding that *G. agilisis* infects *M. paraguayana* in southern Brazil represents a new locality record, which extends the geographical distribution and habitat of this parasite. Cestodes of the family Anoplocephalidae are frequently reported in marsupials of the Neotropical and Nearctic regions (Sandars, 1957; Gomes, 1979; Campbell et al., 2003). Although *M. bivittata*, which is related to *M. paraguayana*, was not identified in the present study, we found specimens of *Pritchardia boliviensis*. *Pritchardia boliviensis* has been described in *Marmosops noctivagus, Metachirus myosuros,* and *Gracilinanus* sp. in the Chaco Biome in Bolivia and Paraguay (Gardner et al., 2013). The findings of the present study expand the parasite distribution area with a new host record, *M. paraguayana.*

The genus *Monodelphis* is one of the most specious among Neotropical marsupials, with seven species recorded in the Southern Cone of South America (Reis et al., 2011). The yellow-sided opossum *M. dimidiata* (Wagner, 1847) is the species of the genus with the southernmost locality register and is classified as a terrestrial insectivore (Paglia et al.,2012). Its distribution includes southern Brazil, Uruguay, and Argentina (Nowak & Walker, 1999; Massoia et al., 2000). There are no reports about the helminth fauna of this species, which may be due to the low population density of this species (Pine & Handley, 2008).

The genus *Viannaia* (Nematoda: Vianaiidae) has been previously reported infecting South American marsupials (Durette-Desset, 1968; Quintão & Costa, 1999; Noronha et al., 2002; Gomes et al., 2003; Byles et al., 2013; Chero et al., 2017; Gentile et al., 2022). *Viannaia hamata* has been described in some marsupial species in southeastern Brazil and in the Brazilian Atlantic rainforest (Pinto, 1977; Gomes et al., 2003; Pinto et al., 2011; Costa-Neto et al., 2019; Gentile et al., 2022). The present study contributes to expanding the distribution of *V. hamata* to southern Brazil.

*Oncicola luehei* (Acanthocephala: Oligacanthorhynchidae) infects the small and large intestines of carnivores and didelphid marsupials across the American continent (Acosta-Virgen et al., 2015; Tavares et al., 2017; Oliveira et al., 2019). It was originally described by Travassos (1917) as a parasite of ring-tailed coatis *Nasua nasua* in Mato Grosso State, Central-Western Brazil. While there are no reports of this parasite in marsupials from Brazil, this acanthocephalan species has been reported to parasitize both *Didelphis virginiana* and *Didelphis marsupialis* in Mexico (Prado Ancona, 1993; CañedaGuzmán, 1997; Acosta-Virgen et al., 2015). This study reinforces the fact that *O. luehei* is a parasite of American marsupials, thereby expanding its geographic distribution and hosts.

*Travassostrongylus* has been reported infecting New World marsupials (Scheibel et al., 2014). *Travassostrongylus sextus* was originally described by Freitas (1937) parasitizing *Mechachirus myosuros* (previously cited as *M. nudicaudaus*) in the state of Rio de Janeiro, Brazil. Our findings represent new host and locality records for this species.

*Trichohelix tuberculata* appears to have low host specificity as previous studies have recorded its occurrence in Tolypeutinae and Euphractinae armadillos, although there has been a report in the skunk *Conepatus chinga* (Travassos, 1937; Lux-Hoppe et al., 2009). The report in skunks may possibly be associated with pseudoparasitism, as skunks are detritivores, and may have acquired *T. tuberculata* when feeding on armadillo carcasses (Peters et al., 2011). The observation of this nematode in *M. dimidiata* may be related to the sympatry allied to the niche overlap of this marsupial with specific hosts (Santos et al., 2019), contributing to the exchange of helminth species and new host adaptation.

A significantly higher prevalence of parasites on male hosts may be attributed to a sex-biased sample, where more male *Marmosa paraguayana* (14 out of 17) were captured compared to females (3 out of 17). Nevertheless, it is presumed that male marsupials are more susceptible to infection due to their more exploratory behavior (Cirino et al., 2020). Sex differences may also be associated with circulating steroid hormones, body mass, size, and heightened physiological stress levels (Klein, 2004).

The absence of a significant relationship between BCI and parasitic burden was also observed in studies with neotropical marsupials and rodents (Püttker et al., 2008; Mota, 2013). This may be attributed to an increased food intake aimed at compensating for the effects of parasitism (Tripet et al., 1997), or it could be due to the fact that the method used to calculate body condition (body mass/body length) has little or no influence on the investigated marsupials (Püttker et al., 2008). It is important to note that the energetic effects of parasitism may not be immediate (Willis & Poulin, 1999), and further studies involving metabolism, food acquisition, or organ masses would be necessary for a better understanding of the influence of nematode infection on the host's condition (Püttker et al., 2008).

The identification of the parasites that infect different species is an essential step towards a more detailed description of the ecology of the host and parasites. The present study represents a new locality record for all helminths described herein, and a new host for four helminth species. According to the authors knowledge, this is the first report of helminth community in *Monodelphis dimidiata*, expanding the knowledge about parasites in marsupials from the Atlantic Forest.
